# Crystal structure of 2-nitro-*N*-(2-nitro­phen­yl)benzamide

**DOI:** 10.1107/S2056989015008695

**Published:** 2015-05-09

**Authors:** Rodolfo Moreno-Fuquen, Alexis Azcárate, Alan R. Kennedy

**Affiliations:** a Departamento de Química – Facultad de Ciencias Naturales y Exactas, Universidad del Valle, Apartado 25360, Santiago de Cali, Colombia; b WestCHEM, Department of Pure and Applied Chemistry, University of Strathclyde, 295 Cathedral Street, Glasgow G1 1XL, Scotland

**Keywords:** crystal structure, benzamide, anti­convulsant properties, anti­microbial properties, inhibitors of diverse enzymes, hydrogen bonding

## Abstract

In the title compound, C_13_H_9_N_3_O_5_, the mean plane of the non-H atoms of the central amide fragment C—N—C(=O)—C [r.m.s. deviation = 0.0442 Å] forms dihedral angles of 71.76 (6) and 24.29 (10)° with the C-bonded and N-bonded benzene rings, respectively. In the crystal, mol­ecules are linked by N—H⋯O hydrogen bonds forming *C*(4) chains along [100]. Weak C—H⋯O contacts link the mol­ecules into (100) sheets containing edge-fused *R*_4_^4^(30) rings. Together, the N—H⋯O and C—H⋯O hydrogen bonds generate a three-dimensional network.

## Related literature   

For anti­convulsant and anti­microbial properties of benzanilide compounds, see: Leander (1992 ▸); Ahles *et al.* (2004 ▸). For studies as selective inhibitors of diverse enzymes, see: Goldman *et al.* (2003 ▸); Weisberg *et al.* (2006 ▸). For related structures, see: Sun *et al.* (2009 ▸); Saeed & Simpson (2009 ▸); Moreno-Fuquen *et al.* (2014 ▸).
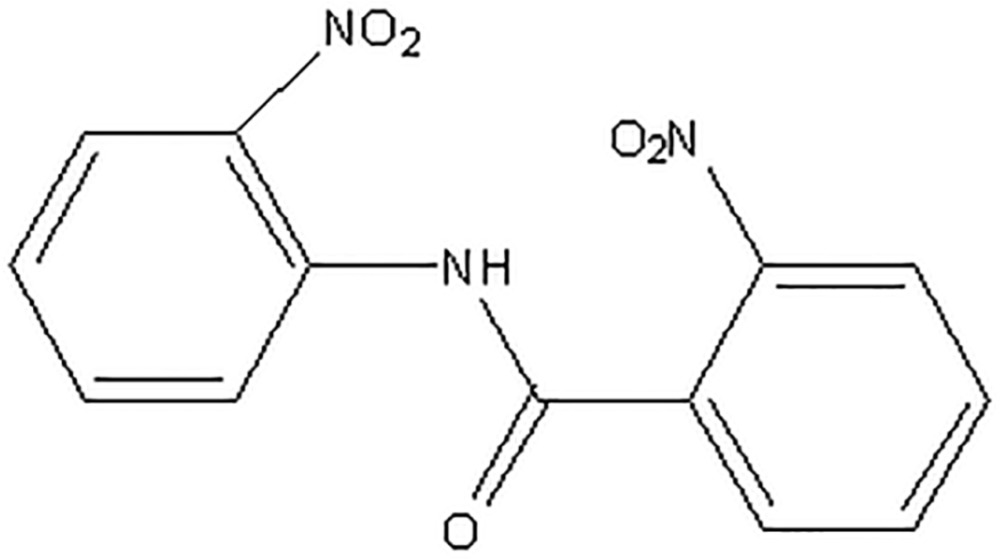


## Experimental   

### Crystal data  

C_13_H_9_N_3_O_5_
*M* *_r_* = 287.23
Orthorhombic, 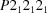

*a* = 7.7564 (2) Å
*b* = 12.1142 (4) Å
*c* = 12.9355 (4) Å
*V* = 1215.45 (6) Å^3^
*Z* = 4
Cu *K*α radiation
μ = 1.06 mm^−1^
*T* = 123 K
0.35 × 0.05 × 0.02 mm

### Data collection  

Oxford Diffraction Gemini S diffractometer
Absorption correction: multi-scan (*CrysAlis PRO*; Oxford Diffraction, 2010 ▸) *T*_min_ = 0.657, *T*_max_ = 1.000
4952 measured reflections
2367 independent reflections
2259 reflections with *I* > 2σ(*I*)
*R*_int_ = 0.019

### Refinement  

*R*[*F* ^2^ > 2σ(*F* ^2^)] = 0.034
*wR*(*F* ^2^) = 0.088
*S* = 1.06
2367 reflections
195 parameters
H atoms treated by a mixture of independent and constrained refinement
Δρ_max_ = 0.20 e Å^−3^
Δρ_min_ = −0.22 e Å^−3^

### 

Data collection: *CrysAlis PRO* (Oxford Diffraction, 2010 ▸); cell refinement: *CrysAlis PRO*; data reduction: *CrysAlis PRO*; program(s) used to solve structure: *SIR92* (Altomare *et al.*, 1994 ▸); program(s) used to refine structure: *SHELXL97* (Sheldrick, 2008 ▸); molecular graphics: *ORTEP-3 for Windows* (Farrugia, 2012 ▸) and *Mercury* (Macrae *et al.*, 2006 ▸); software used to prepare material for publication: *WinGX* (Farrugia, 2012 ▸).

## Supplementary Material

Crystal structure: contains datablock(s) I, global. DOI: 10.1107/S2056989015008695/hb7415sup1.cif

Structure factors: contains datablock(s) I. DOI: 10.1107/S2056989015008695/hb7415Isup2.hkl

Supporting information file. DOI: 10.1107/S2056989015008695/hb7415Isup3.cml

**Fig. 1.**
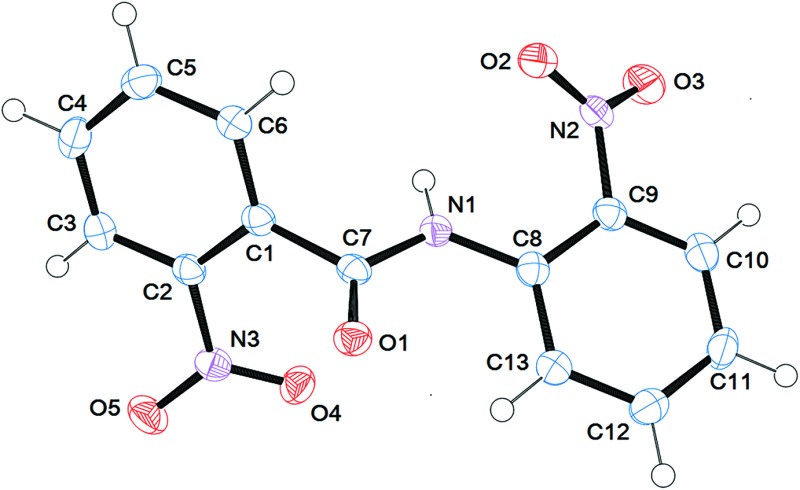
The molecular structure of (I) with displacement ellipsoids drawn at the 50% probability level. H atoms are shown as spheres of arbitrary radius.

. DOI: 10.1107/S2056989015008695/hb7415fig1.tif
The mol­ecular structure of (I) with displacement ellipsoids drawn at the 50% probability level. H atoms are shown as spheres of arbitrary radius.

**Fig. 2.**
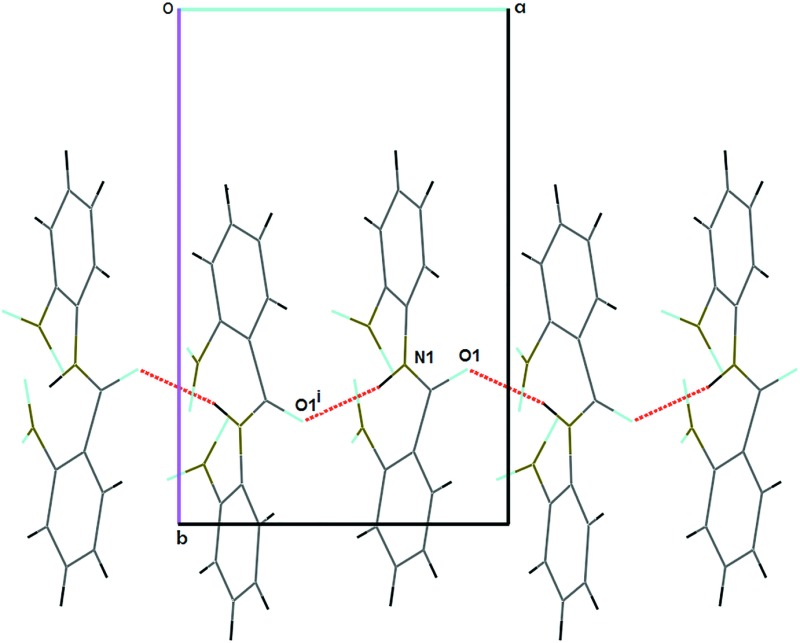
Part of the crystal structure of (I), showing the formation of C(4) chains along [100] [symmetry code: (i) x - 1/2, -y + 3/2, -z + 1].

**Fig. 3.**
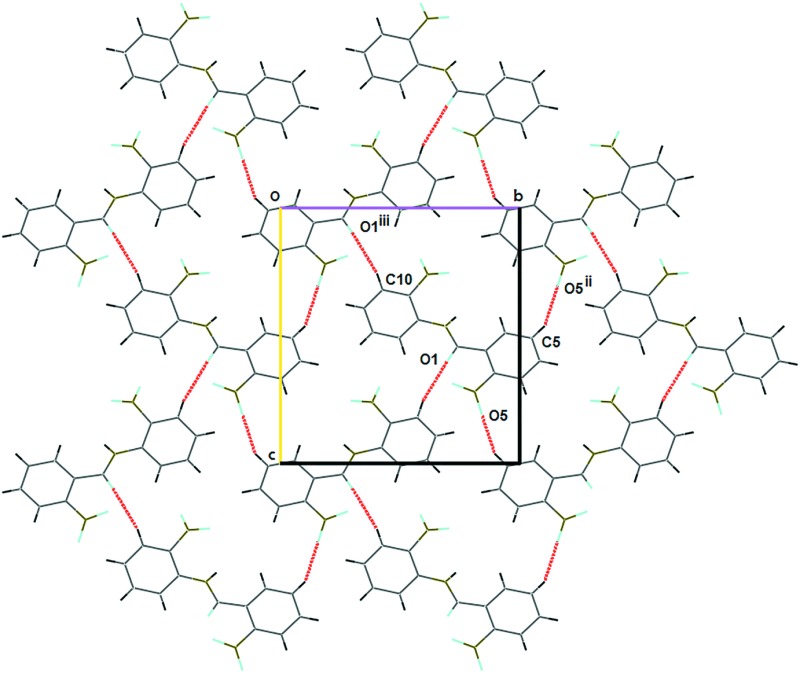
Part of the crystal structure of (I), showing the formation of R44(30) rings within a 2-D hydrogen-bonded network (dashed lines) running parallel to (100) [Symmetry codes: (ii) -x + 3/2, -y + 2, z - 1/2; (iii) -x + 3/2, -y + 1, z - 1/2].

Click here for additional data file.C x y z . DOI: 10.1107/S2056989015008695/hb7415fig2.tif
Part of the crystal structure of (I), showing the formation of *C*(4) chains along [100] [symmetry code: (i) *x* − 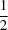, −*y* + 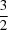, −*z* + 1].

x y z x y z . DOI: 10.1107/S2056989015008695/hb7415fig3.tif
Part of the crystal structure of (I), showing the formation of 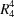
(30) rings within a 2-D hydrogen-bonded network (dashed lines) running parallel to (100) [Symmetry codes: (ii) −*x* + 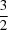, −*y* + 2, *z* − 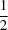; (iii) −*x* + 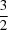, −*y* + 1, *z* − 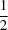].

CCDC reference: 1063243

Additional supporting information:  crystallographic information; 3D view; checkCIF report

## Figures and Tables

**Table 1:** Hydrogen-bond geometry (, )

*D*H*A*	*D*H	H*A*	*D* *A*	*D*H*A*
N1H1*N*O1^i^	0.93(3)	2.00(3)	2.859(2)	154(2)
C5H5O5^ii^	0.95	2.57	3.427(3)	150
C10H10O1^iii^	0.95	2.46	3.271(3)	144

Symmetry codes: (i) 
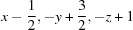
; (ii) 
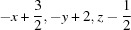
; (iii) 
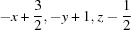
.

## supplementary crystallographic information

### S1. Comment

The crystal structure determination of 2-nitro-N-(2-nitrophenyl)benzamide (I), is part of a study on phenylbenzamides carried out in our research group, and it was synthesized from the reaction between of 2-nitrobenzoic acid and 2-nitroaniline mediated by the presence of thionyl chloride. Benzanilides are versatile intermediate towards a diversity of heterocyclic compounds. Benzanilide systems as ameltolid, very similar to the molecule under study, have different properties ranging from anticonvulsant (Leander, 1992); antimicrobial drug (suramin) or as treatment in patients with prostate carcinoma (Alhes *et al.*, 2004); as inhibitor of tyrosine-kinase, (imatinib) (Goldman *et al.*, 2003) or a selective inhibitor of BCR-ABL, (nilotinib) (Weisberg *et al.*, 2006). Similar compounds to (I) have been reported in the literature: N-(2,4-Dinitrophenyl)-4-nitrobenzamide (II) (Sun *et al.*, 2009), N-(2-Nitrophenyl)benzamide (III) (Saed & Simpson, 2009) and 4-Bromo-N-(2-nitrophenyl)benzamide (IV) (Moreno-Fuquen *et al.*, 2014). The molecular structure of (I) is shown in Fig. 1. The central amide moiety, C8—N1-C7(═O1)—C1, is essentially planar (r.m.s. deviation for all non-H atoms = 0.0442 Å) and it forms dihedral angles of 71.76 (6)° with the C1-C6 and 24.29 (10)° with the C8-C13 rings respectively. Bond lengths and bond angles in the molecule are in a good agreement with those found in the related compounds (II), (III) and (IV). A small lengthening of C7-N1 bond in (III) is observed [N1-C7= 1.3742 (11)Å], possibly caused by the formation of intramolecular S rings (6) in that structure. In the crystal structure (Fig. 2), molecules are linked by N-H···O hydrogen bonds of medium-strength and weak C-H···O intermolecular contacts (see Table 1). The N1-H1···O1 hydrogen bond interactions are responsible for crystal growth in [100]. In this interaction, the N-H in the molecule at (x,y,z) acts as a hydrogen-bond donor to O1 atom of the carbonyl group at (x-1/2,-y+3/2,-z+1). These interactions generate C(4) chains of molecules along [100]. Two C-H···O weak intermolecular contacts are further observed that run parallel to the bc plane in this structure (see Fig. 3). The group C5-H5 in the molecule at (x,y,z) acts as hydrogen bond donor to O5 atom of the nitro group in the molecule at (-x+3/2,-y+2,+z-1/2) and the C10-H10 group in the molecule at (x,y,z) acts as a hydrogen bond donor to O1 atom of the carbonyl group in the molecule at (-x+3/2,-y+1,+z-1/2). The combination of these interactions generate edge-fused R_4_^4^(30) rings.

### S2. Experimental

A mass of 0.200 g (1.197 mmol) of 2-nitrobenzoic acid was refluxed with 2 ml of thionyl chloride for one hour. Then an equimolar amount of 2-nitroaniline was added and dissolved in 10 ml of acetonitrile and it was placed under reflux and constant stirring for 3 hours. Subsequently, the final solvent was slowly evaporated to obtain yellow needles of the title compound. [m.p. 431 (1)K].

### S3. Refinement

All H-atoms were positioned in geometrically idealized positions, C—H = 0.95 Å, and were refined using a riding-model approximation with *U*_iso_(H) constrained to 1.2 times *U*_eq_ of the respective parent atom. H1N atom was found from the Fourier maps and its coordinates were refined freely.

### Crystal data

**Table:**

C_13_H_9_N_3_O_5_	*D*_x_ = 1.570 Mg m^−^^3^
*M**_r_* = 287.23	Melting point: 431(1) K
Orthorhombic, *P*2_1_2_1_2_1_	Cu *K*α radiation, λ = 1.54180 Å
*a* = 7.7564 (2) Å	Cell parameters from 2546 reflections
*b* = 12.1142 (4) Å	θ = 5.0–72.8°
*c* = 12.9355 (4) Å	µ = 1.06 mm^−^^1^
*V* = 1215.45 (6) Å^3^	*T* = 123 K
*Z* = 4	Needle, yellow
*F*(000) = 592	0.35 × 0.05 × 0.02 mm

### Data collection

**Table:**

Oxford Diffraction Gemini S diffractometer	2367 independent reflections
Radiation source: fine-focus sealed tube	2259 reflections with *I* > 2σ(*I*)
Graphite monochromator	*R*_int_ = 0.019
ω scans	θ_max_ = 72.9°, θ_min_ = 6.8°
Absorption correction: multi-scan (*CrysAlis PRO*; Oxford Diffraction, 2010)	*h* = −7→9
*T*_min_ = 0.657, *T*_max_ = 1.000	*k* = −13→14
4952 measured reflections	*l* = −15→15

### Refinement

**Table:**

Refinement on *F*^2^	Primary atom site location: structure-invariant direct methods
Least-squares matrix: full	Secondary atom site location: difference Fourier map
*R*[*F*^2^ > 2σ(*F*^2^)] = 0.034	Hydrogen site location: inferred from neighbouring sites
*wR*(*F*^2^) = 0.088	H atoms treated by a mixture of independent and constrained refinement
*S* = 1.06	*w* = 1/[σ^2^(*F*_o_^2^) + (0.0496*P*)^2^ + 0.2131*P*] where *P* = (*F*_o_^2^ + 2*F*_c_^2^)/3
2367 reflections	(Δ/σ)_max_ < 0.001
195 parameters	Δρ_max_ = 0.20 e Å^−^^3^
0 restraints	Δρ_min_ = −0.22 e Å^−^^3^

### Special details

**Table:**

Experimental. CrysAlisPro, Agilent Technologies, Version 1.171.34.46 (release 25-11-2010 CrysAlis171 .NET) (compiled Nov 25 2010,17:55:46) Empirical absorption correction using spherical harmonics, implemented in SCALE3 ABSPACK scaling algorithm.
Geometry. All esds (except the esd in the dihedral angle between two l.s. planes) are estimated using the full covariance matrix. The cell esds are taken into account individually in the estimation of esds in distances, angles and torsion angles; correlations between esds in cell parameters are only used when they are defined by crystal symmetry. An approximate (isotropic) treatment of cell esds is used for estimating esds involving l.s. planes.
Refinement. Refinement of *F*^2^ against ALL reflections. The weighted *R*-factor *wR* and goodness of fit *S* are based on *F*^2^, conventional *R*-factors *R* are based on *F*, with *F* set to zero for negative *F*^2^. The threshold expression of *F*^2^ > σ(*F*^2^) is used only for calculating *R*-factors(gt) *etc*. and is not relevant to the choice of reflections for refinement. *R*-factors based on *F*^2^ are statistically about twice as large as those based on *F*, and *R*- factors based on ALL data will be even larger.

### Fractional atomic coordinates and isotropic or equivalent isotropic displacement parameters (Å^2^)

**Table:**

	*x*	*y*	*z*	*U*_iso_*/*U*_eq_	
O1	0.8757 (2)	0.69891 (13)	0.59755 (12)	0.0235 (4)	
O2	0.6495 (2)	0.70531 (14)	0.25441 (12)	0.0304 (4)	
O3	0.4638 (3)	0.58573 (16)	0.20009 (14)	0.0363 (5)	
O4	0.5324 (2)	0.71871 (14)	0.68775 (13)	0.0297 (4)	
O5	0.5249 (3)	0.84234 (16)	0.80856 (13)	0.0348 (5)	
N1	0.6852 (2)	0.69018 (17)	0.46280 (14)	0.0215 (4)	
N2	0.5754 (2)	0.61585 (17)	0.26036 (15)	0.0240 (4)	
N3	0.5572 (2)	0.81237 (17)	0.71999 (15)	0.0248 (4)	
C1	0.7127 (3)	0.85927 (19)	0.55862 (17)	0.0207 (5)	
C2	0.6264 (3)	0.89486 (19)	0.64730 (17)	0.0212 (5)	
C3	0.5993 (3)	1.0050 (2)	0.66891 (18)	0.0250 (5)	
H3	0.5410	1.0265	0.7303	0.030*	
C4	0.6585 (3)	1.0837 (2)	0.5998 (2)	0.0282 (5)	
H4	0.6418	1.1600	0.6136	0.034*	
C5	0.7424 (3)	1.0505 (2)	0.51017 (19)	0.0274 (5)	
H5	0.7818	1.1045	0.4623	0.033*	
C6	0.7695 (3)	0.9394 (2)	0.48978 (18)	0.0226 (5)	
H6	0.8273	0.9180	0.4282	0.027*	
H1N	0.607 (4)	0.734 (2)	0.427 (2)	0.030 (7)*	
C7	0.7639 (3)	0.74034 (19)	0.54308 (17)	0.0202 (5)	
C8	0.6909 (3)	0.57706 (19)	0.43817 (18)	0.0214 (5)	
C9	0.6276 (3)	0.5389 (2)	0.34294 (17)	0.0223 (5)	
C10	0.6114 (3)	0.4274 (2)	0.32093 (19)	0.0275 (5)	
H10	0.5633	0.4042	0.2570	0.033*	
C11	0.6657 (3)	0.3505 (2)	0.3926 (2)	0.0294 (5)	
H11	0.6574	0.2738	0.3780	0.035*	
C12	0.7325 (3)	0.3865 (2)	0.4863 (2)	0.0290 (5)	
H12	0.7711	0.3338	0.5356	0.035*	
C13	0.7437 (3)	0.4977 (2)	0.50897 (18)	0.0254 (5)	
H13	0.7882	0.5203	0.5740	0.030*	

### Atomic displacement parameters (Å^2^)

**Table:**

	*U*^11^	*U*^22^	*U*^33^	*U*^12^	*U*^13^	*U*^23^
O1	0.0236 (8)	0.0239 (8)	0.0231 (8)	−0.0009 (7)	−0.0027 (6)	0.0021 (7)
O2	0.0379 (10)	0.0292 (9)	0.0242 (8)	−0.0038 (8)	0.0001 (7)	0.0028 (7)
O3	0.0408 (10)	0.0401 (11)	0.0278 (10)	−0.0036 (9)	−0.0132 (8)	−0.0007 (8)
O4	0.0346 (9)	0.0225 (9)	0.0321 (9)	−0.0046 (7)	0.0051 (7)	−0.0003 (8)
O5	0.0417 (10)	0.0421 (11)	0.0205 (9)	−0.0040 (8)	0.0053 (8)	−0.0024 (8)
N1	0.0251 (9)	0.0206 (10)	0.0188 (9)	0.0031 (8)	−0.0020 (8)	0.0000 (8)
N2	0.0258 (10)	0.0289 (10)	0.0173 (9)	0.0020 (8)	0.0012 (8)	−0.0017 (8)
N3	0.0228 (9)	0.0288 (11)	0.0227 (9)	−0.0009 (8)	0.0014 (8)	0.0017 (8)
C1	0.0198 (10)	0.0231 (12)	0.0191 (11)	−0.0015 (9)	−0.0041 (9)	0.0006 (9)
C2	0.0206 (10)	0.0243 (12)	0.0186 (11)	−0.0014 (9)	−0.0039 (9)	0.0005 (9)
C3	0.0246 (10)	0.0262 (12)	0.0241 (11)	−0.0001 (10)	−0.0020 (9)	−0.0047 (10)
C4	0.0292 (12)	0.0205 (11)	0.0347 (13)	0.0005 (10)	−0.0061 (11)	−0.0037 (10)
C5	0.0284 (12)	0.0254 (13)	0.0285 (12)	−0.0023 (10)	−0.0047 (10)	0.0056 (11)
C6	0.0229 (11)	0.0246 (13)	0.0204 (11)	−0.0002 (9)	0.0003 (9)	0.0001 (10)
C7	0.0205 (10)	0.0238 (12)	0.0164 (10)	−0.0024 (8)	0.0030 (9)	0.0015 (9)
C8	0.0195 (10)	0.0226 (11)	0.0221 (11)	−0.0004 (9)	0.0026 (9)	−0.0013 (9)
C9	0.0210 (11)	0.0255 (12)	0.0204 (11)	0.0027 (9)	0.0029 (9)	−0.0004 (9)
C10	0.0295 (11)	0.0288 (13)	0.0244 (12)	−0.0017 (10)	0.0019 (10)	−0.0052 (10)
C11	0.0354 (13)	0.0186 (11)	0.0341 (13)	0.0006 (10)	0.0024 (12)	−0.0052 (10)
C12	0.0326 (13)	0.0243 (13)	0.0300 (13)	0.0013 (10)	−0.0017 (10)	0.0032 (11)
C13	0.0287 (11)	0.0249 (13)	0.0225 (11)	0.0010 (9)	−0.0022 (10)	−0.0004 (10)

### Geometric parameters (Å, º)

**Table:**

O1—C7	1.225 (3)	C4—C5	1.389 (4)
O2—N2	1.229 (3)	C4—H4	0.9500
O3—N2	1.221 (3)	C5—C6	1.388 (3)
O4—N3	1.224 (3)	C5—H5	0.9500
O5—N3	1.228 (3)	C6—H6	0.9500
N1—C7	1.349 (3)	C8—C13	1.389 (3)
N1—C8	1.408 (3)	C8—C9	1.405 (3)
N1—H1N	0.93 (3)	C9—C10	1.385 (3)
N2—C9	1.475 (3)	C10—C11	1.381 (4)
N3—C2	1.473 (3)	C10—H10	0.9500
C1—C6	1.389 (3)	C11—C12	1.388 (4)
C1—C2	1.396 (3)	C11—H11	0.9500
C1—C7	1.508 (3)	C12—C13	1.382 (4)
C2—C3	1.379 (3)	C12—H12	0.9500
C3—C4	1.386 (4)	C13—H13	0.9500
C3—H3	0.9500		
			
C7—N1—C8	126.7 (2)	C5—C6—C1	120.5 (2)
C7—N1—H1N	115.1 (18)	C5—C6—H6	119.7
C8—N1—H1N	117.5 (17)	C1—C6—H6	119.7
O3—N2—O2	123.7 (2)	O1—C7—N1	125.4 (2)
O3—N2—C9	117.9 (2)	O1—C7—C1	120.1 (2)
O2—N2—C9	118.33 (19)	N1—C7—C1	114.4 (2)
O4—N3—O5	124.1 (2)	C13—C8—C9	117.0 (2)
O4—N3—C2	117.94 (19)	C13—C8—N1	122.2 (2)
O5—N3—C2	118.0 (2)	C9—C8—N1	120.5 (2)
C6—C1—C2	117.6 (2)	C10—C9—C8	122.2 (2)
C6—C1—C7	119.9 (2)	C10—C9—N2	116.3 (2)
C2—C1—C7	122.1 (2)	C8—C9—N2	121.5 (2)
C3—C2—C1	122.6 (2)	C11—C10—C9	119.5 (2)
C3—C2—N3	118.1 (2)	C11—C10—H10	120.3
C1—C2—N3	119.3 (2)	C9—C10—H10	120.3
C2—C3—C4	119.0 (2)	C10—C11—C12	119.2 (2)
C2—C3—H3	120.5	C10—C11—H11	120.4
C4—C3—H3	120.5	C12—C11—H11	120.4
C3—C4—C5	119.6 (2)	C13—C12—C11	121.0 (2)
C3—C4—H4	120.2	C13—C12—H12	119.5
C5—C4—H4	120.2	C11—C12—H12	119.5
C6—C5—C4	120.7 (2)	C12—C13—C8	121.1 (2)
C6—C5—H5	119.7	C12—C13—H13	119.5
C4—C5—H5	119.7	C8—C13—H13	119.5
			
C6—C1—C2—C3	−1.2 (3)	C6—C1—C7—N1	−72.2 (3)
C7—C1—C2—C3	171.1 (2)	C2—C1—C7—N1	115.7 (2)
C6—C1—C2—N3	177.28 (19)	C7—N1—C8—C13	16.9 (3)
C7—C1—C2—N3	−10.5 (3)	C7—N1—C8—C9	−169.1 (2)
O4—N3—C2—C3	157.7 (2)	C13—C8—C9—C10	2.5 (3)
O5—N3—C2—C3	−20.9 (3)	N1—C8—C9—C10	−171.9 (2)
O4—N3—C2—C1	−20.8 (3)	C13—C8—C9—N2	−177.2 (2)
O5—N3—C2—C1	160.5 (2)	N1—C8—C9—N2	8.4 (3)
C1—C2—C3—C4	0.6 (3)	O3—N2—C9—C10	28.9 (3)
N3—C2—C3—C4	−177.9 (2)	O2—N2—C9—C10	−149.3 (2)
C2—C3—C4—C5	0.4 (3)	O3—N2—C9—C8	−151.5 (2)
C3—C4—C5—C6	−0.8 (4)	O2—N2—C9—C8	30.4 (3)
C4—C5—C6—C1	0.1 (4)	C8—C9—C10—C11	−2.8 (3)
C2—C1—C6—C5	0.8 (3)	N2—C9—C10—C11	176.8 (2)
C7—C1—C6—C5	−171.6 (2)	C9—C10—C11—C12	1.2 (4)
C8—N1—C7—O1	12.8 (4)	C10—C11—C12—C13	0.7 (4)
C8—N1—C7—C1	−171.3 (2)	C11—C12—C13—C8	−1.0 (4)
C6—C1—C7—O1	103.9 (3)	C9—C8—C13—C12	−0.5 (3)
C2—C1—C7—O1	−68.2 (3)	N1—C8—C13—C12	173.7 (2)

### Hydrogen-bond geometry (Å, º)

**Table:**

*D*—H···*A*	*D*—H	H···*A*	*D*···*A*	*D*—H···*A*
N1—H1*N*···O1^i^	0.93 (3)	2.00 (3)	2.859 (2)	154 (2)
C5—H5···O5^ii^	0.95	2.57	3.427 (3)	150
C10—H10···O1^iii^	0.95	2.46	3.271 (3)	144

Symmetry codes: (i) *x*−1/2, −*y*+3/2, −*z*+1; (ii) −*x*+3/2, −*y*+2, *z*−1/2; (iii) −*x*+3/2, −*y*+1, *z*−1/2.
