# Supplementary figures and images for: Crystal structure of 2-nitro-N-(2-nitro­phen­yl)benzamide
Source: Acta Crystallogr E Crystallogr Commun. 2015 May 9;71(Pt 6):o389–90. doi: 10.1107/S2056989015008695 (PMC4459296; doi:10.1107/S2056989015008695)

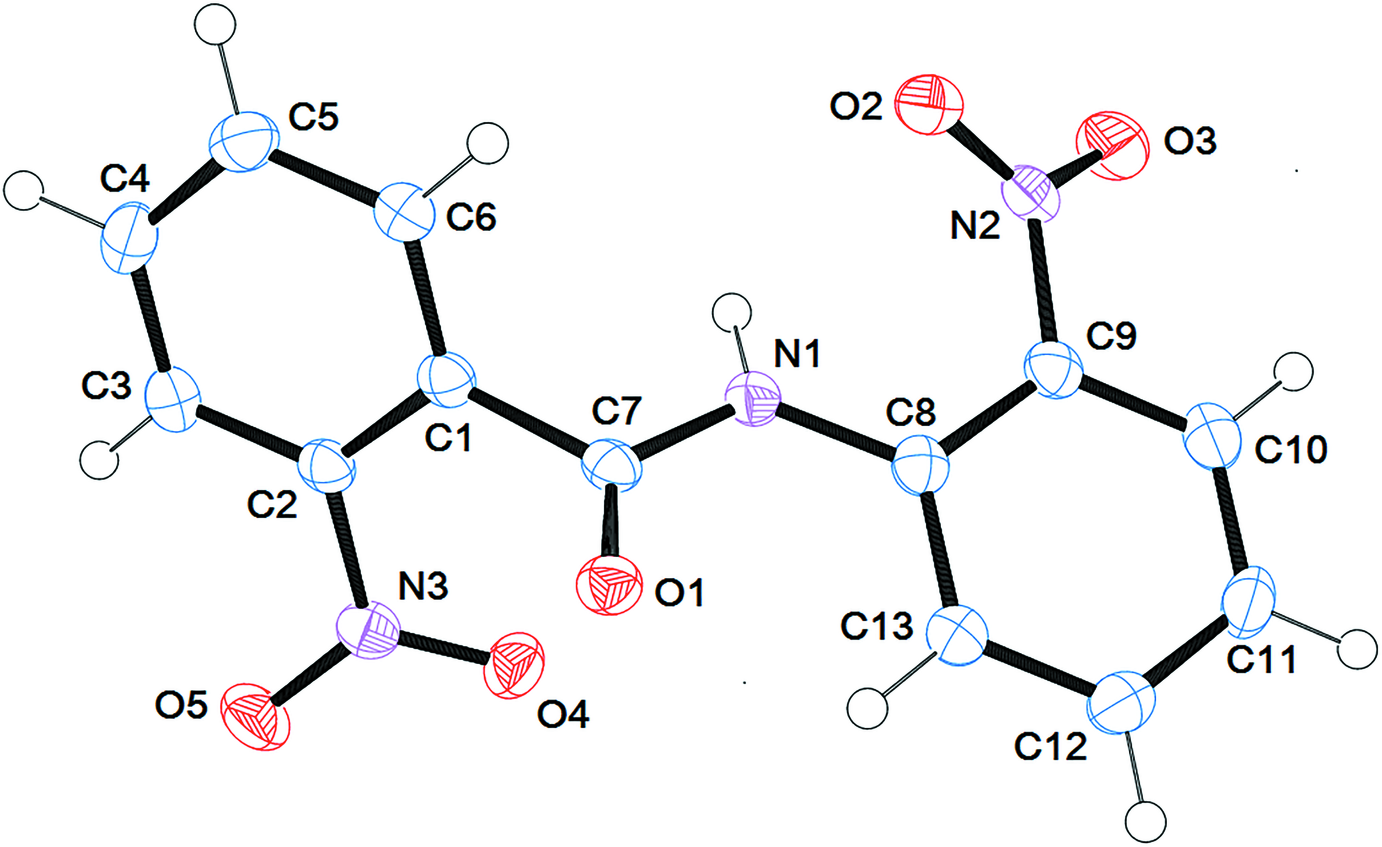

Supplement: Supplementary file 4 [file e-71-0o389-fig1.tif]

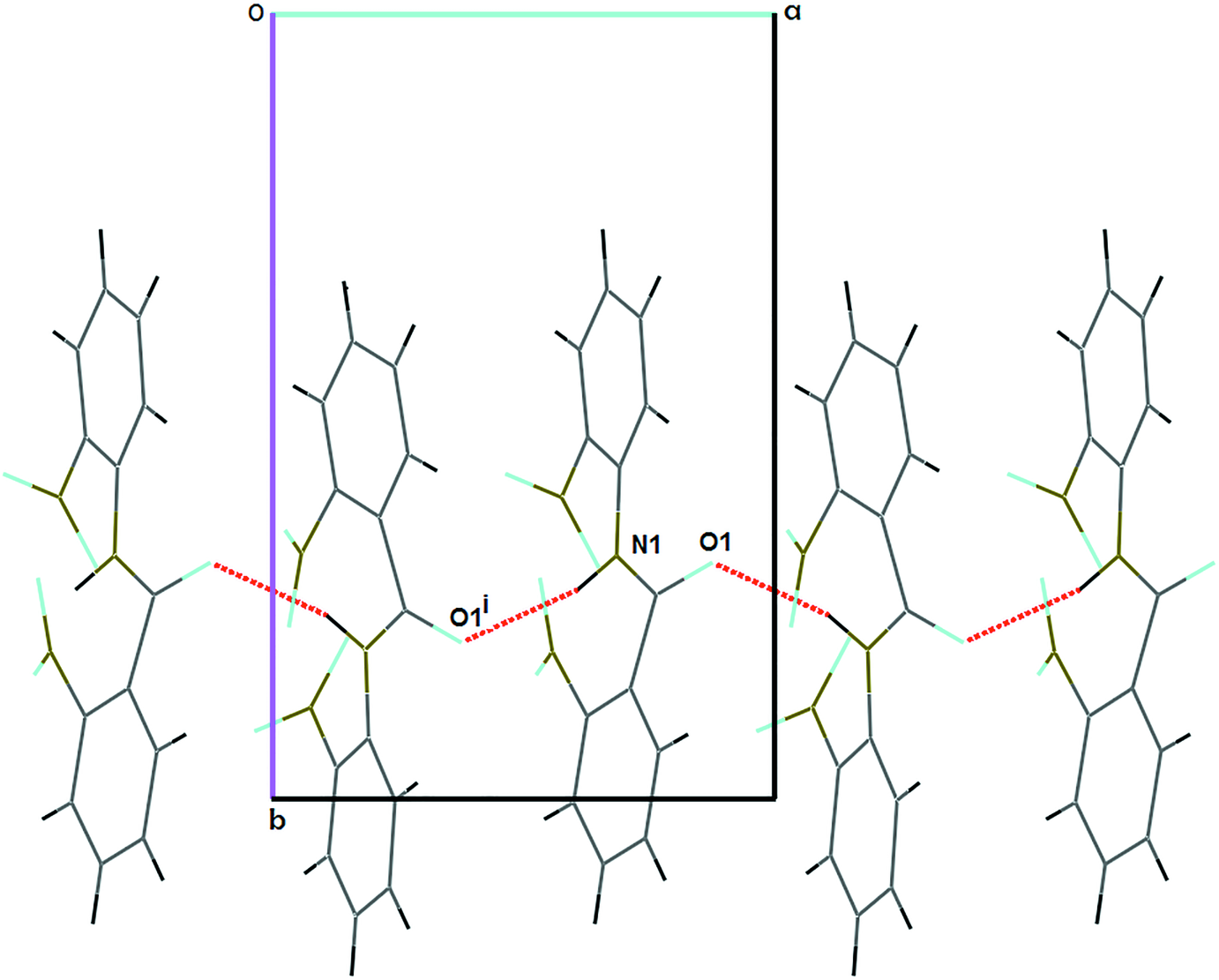

Supplement: Supplementary file 5 [file e-71-0o389-fig2.tif]

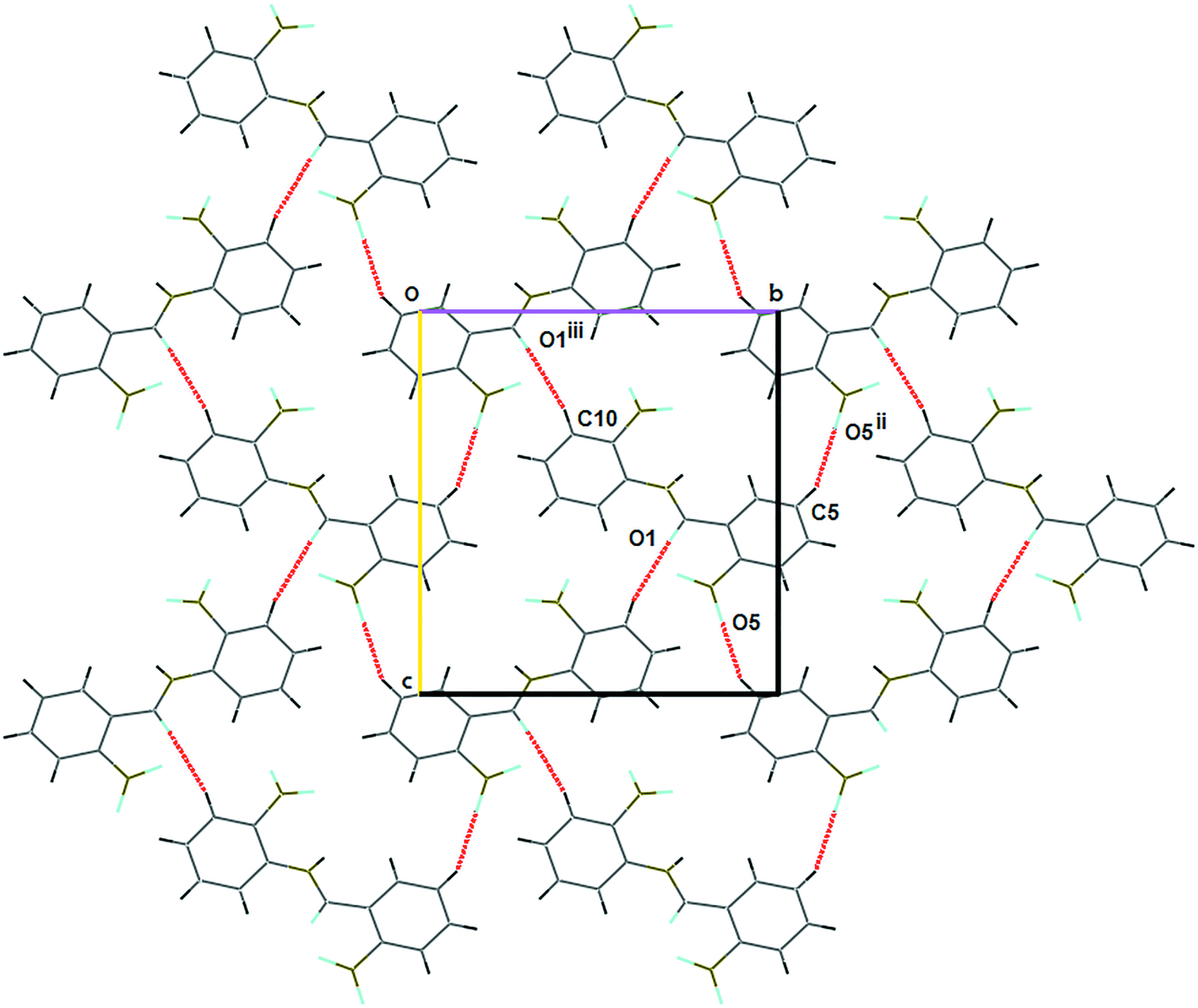

Supplement: Supplementary file 6 [file e-71-0o389-fig3.tif]
